# Organising the self-employed: combining community unionism, coworking and cooperativism across contexts

**DOI:** 10.12688/openreseurope.15798.1

**Published:** 2023-05-16

**Authors:** Frederick Harry Pitts, Paolo Borghi, Annalisa Murgia

**Affiliations:** 1 Department of Humanities and Social Sciences, University of Exeter, Exeter, England, UK; 2 Department of Economics ‘Marco Biagi’, Universita degli Studi di Modena e Reggio Emilia, Modena, Emilia-Romagna, Italy; 3 Department of Social and Political Sciences, Universita degli Studi di Milano, Milan, Lombardy, Italy

**Keywords:** Community Unionism, Cooperativism, Coworking, Organising, Self-Employed

## Abstract

The growing insecurity, flexibilisation and fragmentation of labour markets goes hand-in-hand with the decrease of social protection levels and collective representation for workers in non-standard employment relationships, such as the hybrid category of ‘solo self-employed workers’. In response, on the one hand, trade unions attempt to approach and organise this heterogenous category of workers. On the other, new freelancer organisations are emerging to improve worker rights and safety, and overcome their social and professional isolation. Reporting the findings of long-term, slow ethnography, we describe a failed collaboration between three new collective actors in the representation and organisation of self-employed workers. In the second half of the 2010s, two UK organisations, Coworking (all names pseudonyms), a coworking space operator working in a deprived ex-industrial area, and Union, a former industrial union, created Coworking.Union, a cooperative trade union offering services and advocacy for the self-employed. Coworking.Union collaborated with Cooperative, a freelancer cooperative based in Northern Europe, with a view to emulate aspects of its model in the UK. We present a detailed reconstruction of the interactions of the three actors over time, including their context, expectations, and visions, starting from the motivations that generated the first contacts, through to the development of operational agreements, up to the failure of these agreements as relations cooled. The case study, and the failed experiment it captures, constitutes an important opportunity to understand the dynamism, complexity, and contradiction manifest in organising the self-employed. While the strategic ingredients of significant organisational innovation were in evidence between the three actors, it generated instead a failure. The case study thus demonstrates the importance of an in-depth analysis of failed attempts at organising the self-employed and their meaning for broader struggles by old and new actors to alter the terrain of the hybrid areas of employment more generally.

## Introduction

The grey zones between self-employment and employment, of precariousness and flexibility produced by contemporary labour markets ( Bozzon & Murgia, 2022; Pitts, 2016; Supiot, 2001) and the ‘non-standard’ ( Burchell *et al.*, 1999; Olsen & Kalleberg, 2004) or ‘alternative’ ( Spreitzer *et al.*, 2017) work arrangements incubated by the digitalisation of the labour process ( Pitts, 2013), demand new organisational forms to represent those excluded by the association of established labour movement actors through the divisions of industrial production. Since the 2000s, unions have sought to reconfigure themselves, and new actors have arisen to connect with broader spatial and geographical communities beyond the traditional workplace. Attempts to reach out to new groups of workers amount to an important source of renewal and growth for unions otherwise struggling to adapt to the new world of work ( Hyman & Gumbrell-McCormick, 2017; Meardi *et al.*, 2021).

Self-employed workers are one such site of potential renewal. Countries like the UK have witnessed dramatic increases in the number of self-employed workers since the 2008 financial crisis, many of them sole traders rather than entrepreneurs with employees of their own. This workforce suffers from low income, insecurity, and under-regulation of so-called ‘false’ or ‘bogus’ self-employment ( Buschoff & Schmidt, 2009; Conen & Schippers, 2019). The diversity of this workforce across classes, sectors, ages, geographies, and professions creates barriers to organisation ( Lockey, 2018), but in recent years common legal and financial issues like rights to employment protections, access to welfare and pensions, late payments from clients and income volatility have been identified as a possible basis to articulate coalitions between self-employed workers ( Taylor, 2017).

Reporting the findings of long-term, ‘slow’ ethnography ( Almond & Connolly, 2020), this paper describes and analyses a failed collaboration between three new collective actors in the representation and organisation of self-employed workers. In the second half of the 2010s, two UK organisations, Coworking, a coworking space operator working in cities and towns in a deprived ex-industrial area, and Union, a former industrial union, created Coworking.Union, a cooperative trade union offering services and advocacy for the self-employed. Coworking.Union then reached out to Cooperative – operating across Northern Europe – with a view to emulate aspects of its model in the UK. Cooperative is a freelancers’ cooperative. Freelance cooperatives of this kind aim to improve the social protection of freelancers while preserving the autonomy in running their business ( Bajard & Leclercq, 2019; Bureau & Corsani, 2017). These organisations also provide freelancers with new services, such as a salary guarantee fund, adapted insurances, advice, training, and legal expertise, and with discounts or free access for members to coworking spaces within or close to their own premises ( Martinelli *et al.*, 2019; Mondon-Navazo *et al.*, 2022).

## Literature review: communities, coworking spaces and cooperatives

The collaboration at the heart of the case study brought together three distinct kinds of new labour market and industrial relations actors that were formed in response to the alternative work arrangements: community unions, represented by Union; coworking spaces, represented by Coworking; and a cooperative providing social protection, represented by Cooperative. In analysing a highly experimental case study, this paper intends to combine different strands of literature that traditionally develop on different planes, while dealing, from different perspectives, with the process of collective organisation of the self-employed: the literature on workers’ representative organisations (in this specific case with a focus on community unionism), the literature on coworking spaces, and that on co-operatives.

### Community unions

Trade unions are renovating their repertoires of contention through coalitions with social movements and new actors both within and beyond national borders ( Ibsen & Tapia, 2017). In particular, this has incorporated attempts on the part of established unions to protect the growing number of unrepresented outsiders ( Hyman & Gumbrell-McCormick, 2017; Meardi *et al.*, 2021) in professional contexts, like advanced tertiary and platform work, where unions have not been present ( Borghi *et al.*, 2021; Joyce *et al.*, 2022).

In the UK, ‘business unionism’ focused on firm-level partnership, narrowly economic concerns and traditional union activities like wage negotiation and bargaining has been challenged by ‘community unionism’. Instigated by the Trades Union Congress’s ‘New Unionism’ campaign and ‘Organising Academy’ ( Heery, 2002; Mollona, 2009), community unionism sought to steer unions away from servicing basic demands to become active in organising the unorganised and effecting wider social change ( O’Grady & Nowak, 2004). In particular, it focused on empowering groups outside the union fold through a spatial rather than skill-based approach to organising across a geographical or professional terrain that transcends the shopfloor ( Heery, 2002; Symon & Crawshaw, 2009). This is seen as relating specifically to flexibilised and precarious workers like the self-employed ( Wills, 2001).

Scholars note a tension in community unionism insofar as it can be perceived as embracing excluded groups only to use the energy produced to power the growth and renewal of the union as a more conventional, traditional industrial relations actor ( Heery, 2002). This tension is seen as being overcome in a further development away from business unionism, ‘social movement unionism’, which radically integrates unions as equal partners with wider struggles beyond the economic and political terrain of the labour movement ( Waterman, 2008).

Union, a leading example of community unionism in the UK, formed in the early noughties out of the remains of industrial unions whose memberships dwindled following the severe decline of those industries. Union has attempted to transcend their original composition to meet the challenges of new labour circumstances, embracing formerly excluded groups and even retraining workers so that they can take work in the expanding service sector. Broadening its focus beyond the declining trades and industries themselves, it turned its attention to all those workers impacted by the deindustrialisation of the communities they represented. This included seeking to represent the self-employed. At the time of the research, Union had sought for some time to follow other organisations in developing a digital platform to offer a variety of services including debts factoring, to ensure that members are paid on time.

### Coworking spaces

Coworking spaces offer desks, resources, and networking opportunities to independent or remote workers of various kinds. Most operate, whether for-profit or not-for-profit, through leasing desks on a daily, weekly, or monthly basis. Some spaces pursue a social mission or serve a specific community ( Butcher, 2018), including by collectivising the often atomised and precarious experience of self-employed work ( Pitts, 2017) by acting as ‘infrastructure for mutual aid’ and a ‘platform of collective action’ ( de Peuter *et al.*, 2017, p. 689). Examples include spaces offering access to health insurance, mutual funds for sickness, maternity leave, and childcare to members, as well as forums for discussion and activism around freelancer rights. Moreover, by ‘fulfil[ling] broader social and economic community development goals in neighbourhoods’ ( Merkel, 2019: 539), some coworking spaces have shown a willingness to address ‘weighty societal challenges’ more widely ( de Peuter *et al.*, 2017, pp. 689, 694), for instance by providing dedicated space and support to women and ethnic minorities ( Grazian, 2020).

However, membership churn, the imbalance between regular dues and volatile income, and cycles of urban regeneration and redevelopment threaten this supportive and socially oriented function. At the same time as increasing the corporatization of the sector, coworking spaces are thus increasingly exploring cooperative business models as a means to weather these tendencies and reproduce the conditions for organising alternatives in this area ( de Peuter *et al.*, 2017). This includes sharing the proceeds of work members to compete for clients collectively, rather than individually, creating safety nets for self-employed workers ( Gandini & Cossu, 2021). Houtbeckers (2018) notes the need for further research explicitly exploring the paradoxes and imperfections inherent in the model.

Founded in 2010, the organisation Coworking is an example of where a cooperative business model has been used to advance a broader social and collective project with the context of coworking spaces. Like Union, Coworking’s origins lie in deindustrialised areas in a specific region of the UK where many of its coworking spaces are located, giving the self-employed and entrepreneurs access to local workspaces. At the beginning of this research, it had over 20 spaces with some 200 members. Prior to the research, Coworking was developing plans to go beyond space provision to offer invoice-factoring and payment-chasing services to members. They were also engaged in advocacy and representations about the upcoming implementation of Universal Credit in the UK, its introduction of a monthly ‘Minimum Income Floor’ governing welfare eligibility being taken to exclude self-employed workers subject to income volatility ( Work & Pensions Committee, 2018).

### Cooperatives providing social protection

Over the past decade or so continental Europe has seen the emergence of a number of innovative experiments in organising and representing independent workers beyond unions. Cooperatives and other forms of mutual aid provide services and protections to the self-employed, including bookkeeping, legal advice, sickness support, access to benefits, and cheap loans ( Bajard & Leclercq, 2019; Mondon-Navazo *et al.*, 2022). Of particular interest are the cooperatives that allow sharing of resources and improving access to social welfare programmes ( Martinelli *et al.*, 2019; Pitts, 2020).

While these innovations are ‘the result of a double and contradictory process of flexibilization and stabilization of workers’ ( Bureau & Corsani, 2017, p. 62), a number of organisational attempts across Europe have been implemented to counteract individualised forms of work and to propose an alternative to self-employment for freelancers ( Mondon-Navazo *et al.*, 2022). In the French context, a new cooperative concept was developed in the mid-1990s: the Business and Employment Cooperatives ( Bureau & Corsani, 2017). In particular, Coopaname ‘offer a system of ‘employment’ to effectively self-employed individuals, so that their social security status, and therefore access to benefits is maintained at the same level as a conventional employee. They do this by paying a salary to the individual member based on their earnings… and pay…PAYE and NI on their behalf’ ( Conaty *et al.*, 2018, p. 37). Assisting with ‘salary smoothing’ and mediating relationships with the welfare and benefits system, these cooperatives receive government support in some contexts as a means to respond to the regulatory problem posed by the growth of the self-employed workforce, with both the Department of Work and Pensions and the government-commissioned Taylor Review of Modern Working Practices displaying an interest in applying this cooperative model in the UK ( Conaty *et al.*, 2018; Taylor, 2017).

Originally founded in the late nineties, Cooperative is one such example. It is ‘a freelancers’ cooperative that developed a novel model to empower freelance creative workers – both commercially and socially – in an attempt to support their careers and to create new forms of solidarity, despite the general trend of a lack of social protection rights and collective representation for the self-employed ( Murgia *et al.*, 2020). Around the time of the research, Cooperative was starting discussions with trade unions about potential collaborations.

## Case study

The research was conducted over a four-year period from 2017 to 2021. The long-term, slow comparative approach (see Almond & Connolly, 2020) employed incorporated participant observation and ethnography at meetings and other events involving representatives from Coworking, Union and Cooperative, in mainland Europe and the UK (the names of organisations have been replaced with pseudonyms). This largely took place in the first phase, spanning 2017–2019. This was complemented with six semi-structured interviews with stakeholders from the three organisations over the course of 2020–2021. Quotes are not attributed to specific participants in the Findings section to protect anonymity. The data generated included field notes and interview transcripts, which were coded inductively by the research team and used to inform the findings presented here. Written informed consent for publication of the participants details in pseudonymised or aggregated form was obtained from the participants.

While much has been written about these three models individually, the uniqueness of the case study presented in this paper is that it represents an attempt to create a new institution from elements of each. The imperfection and ultimate failure of this experiment has much to tell us about the paradoxes and problems that characterise them individually.

### Foundations and motivations

***Union.*** Within the UK trade union movement precariousness has been an elusive issue for a long time; it ‘was never raised as a concern’, according to an interviewee from Union. This means that the labour movement has not found itself ‘in the place it could potentially have been’. Likewise, self-employment was not seen as a positive career and lifestyle choice for workers. Self-employed workers have sometimes been seen as ‘somehow undermining traditional employment’, rather than adhering to the adage that ‘a rising tide lifts all boats’, a principle the participant thought was important to secure the broadest array of workers ‘access to rights at work’. This orientation guided Union’s future strategy for the union’s development, against a backdrop of the ‘decline’ and ‘hollowing out’ of its former areas of industrial strength. The rise of self-employment was indeed an opportunity to ‘accumulate’ a ‘new generation’ of workers defined both by the nascent gig economy and new professional identities, and sometimes by a mix of both between which union membership would need to follow.

From the early noughties onwards, Union had established a dedicated Assistant General Secretary role, appointed by the union’s executive, with responsibility for the development of strategy and policy, engaging government, other unions and new organisations and social movements through a specialised research and communications team. As one interviewee explained, this period saw Union explore the possibility of branching out into the representation of self-employed workers, including through a potential partnership with a union for workers in construction, where various forms of self-employment are widespread. The construction union was seen as a basis for more extensive organisation of the self-employed, but when it became part of another larger union in the second half of the 2010s, this opportunity vanished. Like construction, broadcasting was also seen as an area where a large number of contracted self-employed workers were co-located in workspaces in which unions could organise to represent them and establish collective bargaining with contractors. The organisation had conversations with a union for workers in film and television prior to the latter joining another larger union, who now bargain on behalf of tens of thousands of self-employed workers in those industries.

These early forays were unsuccessful, but a sense of direction was nonetheless set for Union to explore further in-house options for expanding into ‘other sectors where your unions are not organized, where self-employment is starting to pick up’. The union increasingly saw the provision of services and offers to potential members as a key way to organise the unorganised in this occupational segment. Union began actively ‘looking for partners’ to collaborate on this more platformised vision of what the union could do. They sounded out collaborators in the corporate world and, crucially, the third sector of cooperatives and social enterprises.

***Coworking.*** Coworking initially started out providing coworking space to self-employed people in cities, towns, and other communities in a deprived ex-industrial area of the UK. Prior to becoming a cooperative in the form of a Community Benefit Society, it was a Community Interest Company, placing constraints on its financial activities. Coworking began in the period immediately following the financial crisis when its founder occupied an office space in a building with many underutilised floors and spaces, persuading the manager to allow him to operate a portion of the unused space as coworking provision. In the latter part of the noughties the space was kitted out and desks rented, initially for free and later for £10 a day, membership doubling each month for six months through word of mouth alone rather than intensive marketing. This experience gave rise to the repeated mantra of one participant, who remarked that ‘you can’t build a community, the community builds itself’.

As spaces proliferated, the burden became greater on the founder to perform ‘thankless tasks’ such as servicing printers, and so Coworking began to build up the capacity of space users themselves to self-organise their community in the workplaces they occupied. This was perceived as challenging the dominance of a ‘start-up’ mentality among coworking spaces, which one interviewee described as resembling ‘youth clubs… full of… boys with their toys thinking they’re gonna build the next new start-up’.

This was challenged not only by Coworking’s greater gender balance, but by the increasingly politicised direction in which it was travelling. This initially sprang from a critique of the ‘agglomeration economics’ by which the main local city related to its nearby rural or industrial ‘hinterland’, and by which the region as a whole related to a UK economy based around London. Their underpinning purpose was combatting loneliness and isolation among independent workers in the communities in which they became active and enabling entrepreneurial workers to remain embedded in the communities in which they live rather than being forced to relocate to metropolitan centres. On a non-profit basis, the expansion of Coworking outwards from the main local city into the wider region as a whole was supported by funding from local government, among other bodies. This financed the establishment of lower-cost workspaces in towns and villages in the region. But they also opened spaces further afield, such as in the Greater London suburbs. An operator of a space incorporated into Coworking described how the relationship worked:

My impression of them was that they were quite different from many of the other places that rent out desks. Most places where you rent a desk out, it's sort of… it's quite sharp. You know, it's very expensive. There's not much… there's no ethos. It's just about people taking over a building of some kind to turn a quick profit. On the contrary [Coworking] seemed to actually genuinely have a connection with, you know, ideas… They were not in any way interfering with our ideas of what we wanted to do in the space, they were just allowing us to do almost whatever we wanted. […] And it was never about trying to create this sort of perfect office, you know, like WeWork type of space, so they were cool.

From this basis, Coworking eventually positioned itself openly, and politically, against a ‘WeWork’-style ‘start-up culture’ and the coworking ‘goldrush’ associated with it, seeking to present a grassroots alternative. The mix of people using their spaces – ‘architects, writers, illustrators, graphic designers, web developers’ – helped reveal the commonalities and continuities in terms of the challenges they faced and ‘the things that are wrong with their lives because of being self-employed’. The realisation that people were in difficult conditions, plus the feeling that ‘no one was even trying to address’ these issues, jolted the organisation out of a period where ‘for a number of years… we were trying to do things that we thought were good without a realist, without a strategic direction’. They then began to become interested in how some trade unions like Equity and the Musicians Union had organised precarious workers in situations of employment and self-employment largely atypical of the profile of trade union members in the UK. However, they maintained a scepticism about the capacity of ‘general unions’ to ‘get’ the experiences and conditions of self-employed workers such as those using Coworking’s spaces.

***Cooperative.*** Sometimes operating against the objections and perceived interests of trade unions in continental European countries, Cooperative was founded to respond to the need among freelance creatives to manage the process of signing contracts with clients and to handle varying patterns of payment, which complicated access to rights and benefits through the state. The creation of a piece of software – later an online platform – solved this issue by automatically creating contracts and cost centres for jobs. The subsequent development of Cooperative offered a wider range of services, including social security arrangements, debt collection, guaranteed monthly income payments, insurance provision, advice on intellectual property, facilitation of loans and late-payment chasing. The platform enables users to employ others, move money between projects, draw down royalties and other payments, arrange transfer of ownership, pay VAT, and set up temporary wrappers for collaborations with other users. Rather than individuals invoicing clients, Cooperative invoices them and then pays the worker business income in the form of a salary through a PAYE-style system. In exchange for a percentage of the revenue, it chases payments and guarantees salary through a mutual guarantee fund. Any user who processes three contracts through the platform must then become a permanent Cooperative ‘worker’, completing timesheets in exchange for employment status, a salary, and the rights and benefits that follow. Tens of thousands of users had registered for the platform. Much of this growth occurred under the radar of the state, occupying the interstices of a weak regulatory environment that had been vacated by trade unions uninterested in the representation of the self-employed.

Prior to the period of the research, Cooperative had been engaged in a process of rapid international expansion within continental Europe that was just slowing down as wariness set in following some costly unsuccessful ventures. This expansion occurred with the support of EU bodies on cultural mobility, and effectively navigated the differences in employment status and benefit provision in different countries by tweaking the online platform to produce the right information for the regulatory context.

Cooperative’s internationalization strategy gradually shifted from active to passive. In the early 2010s, Cooperative was ‘looking eagerly to develop internationally because… our main objective was to support mobility of artists’. ‘Ambassadors’ would be found in other countries who were willing to set up a branch, and the feasibility evaluated on the basis of ‘labour law, fiscal law, social security and taxation’. There was also consideration of the ‘cultural aspects’ around whether people were ‘eager to become salaried or more into the entrepreneurial kind of vibe’. In general, the less ‘structured’ the field of self-employment in a given context, the easier it was to implement the Cooperative model: ‘if there’s a no man’s land, [it] is usually easier to move’.

In the first half of the 2010s, Cooperative had an ‘ambassador’ active in the UK, but by the middle of the decade it had become clear that it was unworkable due to a combination of personality, poor fit of the partner, and more substantive ‘socio-economic, legal and cultural’ issues, according to one interviewee. These included that the UK did not seem to have ‘much of a big difference between self-employment and salaried workers’, enhanced social protection was seen as ‘not very attractive’ to the target population, and a perceived ‘individualistic approach’ stymied the promotion of the collective aspect of the Cooperative model. ‘Internationalization’ having been voted last place in the Cooperative General Assembly’s ballot of priorities, at the time the research began, Cooperative was moving to a less ‘eager’ position vis-à-vis opportunities to internationalize, even though external interest was increasing for exporting the model elsewhere.

### Connections and collaboration

Despite their relaxed reticence about further exploration of opportunities to extend Cooperative in the UK and elsewhere, conversations with a research, policy and advocacy organisation for British cooperatives persuaded Cooperative that perhaps the UK context was somewhat less ‘individualistic’ than they had initially imagined. A report published by the research, policy and advocacy organisation had featured Cooperative as a case study in new frameworks for representing the self-employed, and the organisation was invited to address the organisations conference. Coworking and Union had both become aware of Cooperative through the report, and Union had in turn become aware of Coworking through the same route. The authors of the report had drawn attention to the potential affinities between the Cooperative model and some of the things Coworking was experimenting with, as well as the broader potential for cooperatives and unions to work together in organising the self-employed. This led to ‘matchmaking’ by the research, policy and advocacy organisation, who convened two conversations: one between Coworking and Union, and another between Coworking and Cooperative.

In the first conversation, Union visited Coworking’s headquarters in the UK, at a time when the latter was still transitioning to a cooperative. From Coworking’s perspective, ‘there were people in Union at the time who saw an opportunity to work with self-employed people… that was… [from] a strategic vision perspective, a good starting point’. Union, meanwhile, perceived in Coworking ‘shared views on how benefits needed to evolve’ in order to support self-employed workers, but was concerned about the capacity to take leaders and members of their union ‘on the journey’ of the collaboration, especially in terms of financial investment and the gains the union would expect in return. One of the key ‘synergies’ was Coworking’s origin in a deprived ex-industrial area, described as ‘as a big tick in the box, given the history of Union in the…industry there’. This eventually produced ‘a very deliberate decision to engage with Coworking’ further.

The second, later, conversation brought Coworking and Cooperative together. In the view of one participant, there was an understanding among those who convened the initial meeting that Coworking was of potential interest to Cooperative because it had begun working in a positive fashion with a trade union where Cooperative had typically struggled ‘with getting the unions to have a conversation with them’. Coworking and Cooperative were doing ‘complementary things… from a different angle’, but nonetheless ‘shared a lot of views on what was going on’, according to the same participant. Likewise, Cooperative saw in Coworking ‘the same purpose’ and the ‘same values’: ‘we want to fight for the same type of world’, as one interviewee put it. While they were not actively seeking a UK venture, preferring to ‘plant seeds in different places’ pending a fully worked-out plan from a potential partner, there was sufficient enthusiasm on Cooperative’s part that they ‘thought it could be really interesting to work the three of us together’, with Coworking and Union. The latter two organisations, over this period, came to see the Cooperative model as a means to structure their own collaborative enterprise in the UK.

***Coworking.Union.*** Coworking.Union, as it was called, represented a digital complement to Coworking’s historically ‘analogue’ foundation. In collaboration with Union, a platform was established to give members access to financial and legal support and advice, as well as providing a basis to project a campaigning voice for the representation of self-employed workers. In particular, Coworking.Union leveraged more extensive financial and organisational resources to afford members invoice-factoring services that would usually be available only to bigger companies – allowing self-employed workers to more effectively chase late payments from clients, which was perceived as one of the ‘biggest things’ confronting self-employed workers:

If you raise a thousand pounds for a project and your clients don’t pay you, what you do? Do you go to your lawyer? It's impossible if you want to stay in the market. Most of the time probably you give up all the time and with that thousand pounds maybe 10% of your annual income…

Over a year prior to the research, Coworking had made the transition from a Union Interest Company – in other words, a social enterprise – to a Union Benefit Society, more suitable to the aims and purposes of a cooperative, with shares of equal value and one member (or shareholder) to one vote. It was technically on this basis that Union became a stakeholder in the organisation. However, locally, there was little engagement in the AGMs, with only 14 of 600 members recorded as attending at one point in the development of the cooperative. At its peak during the period of research, Coworking.Union had some 800 members, many of whom were not users of Coworking’s coworking spaces. However, around 90 per cent of Coworking’s space users were members of Coworking.Union.

Coworking.Union cross-subsidised the provision of coworking space insofar as the ‘connectors’ who acted as ‘organisers’ for the organisation were charged not only with recruiting members for the platform but also locating spaces from which Coworking could rent desks. This included those in existing coworking spaces where Coworking could sublet one or two desks as a foothold to expand reach and membership. In this way the two – Coworking and Coworking.Union – were conceived as cross-subsidising one another insofar as desks translated into members and members into desks, and, during the period when Coworking.Union was taking off, there was certainly a quickening of the pace at which new spaces or desks were coming on stream. Coworking had used the connections opened by Union to open spaces in atypical coworking locations like Oldham and Bury on the outskirts of Greater Manchester.

The main champion of the collaboration within Union departed the union for a new position elsewhere very soon after the relationship with Coworking was formalised. While one interviewee from the union called this ‘the honeymoon period of the marriage’, it was not without tensions. Whilst Coworking were well aware of the union’s ‘strategic need to arrest the decline in membership’ by reaching out to a new cohort of workers, there were still early signs that the union’s focus on ‘growing membership quicker’ as a means of ‘showing return on [their] investment’ ran counter to Coworking’s aspirations for the relationship.

One area in which both parties were in close alignment was in the aspiration to establish themselves as what one participant from Union called ‘the voice for self-employed workers’, which both organisations understood as entailing growing the membership, because, as an interviewee from Coworking suggested, ‘the more members you got the stronger your voice is’. The question of autonomy cropped up early on insofar as Union understood the organisations as separate actors in this space who were engaged in a partnership, as opposed to projecting one voice together. ‘My view was that we were setting out on the same path, same trajectory, just at a different pace’, as one Union official put it.

For Union, Coworking.Union was a means by which to establish ‘credibility’ in this space among both self-employed workers and government by growing the organisation’s membership. With these foundations in place, it would be possible to ‘start to influence policy and political direction, whether it’s in the Labour Party or the TUC, or, more broadly, in your engagement with government ministers’. Meanwhile, Union’s support enabled Coworking to take steps towards realising its longstanding ambition to represent a political voice for self-employed workers. In their new ventures into poorer post-industrial communities elsewhere in the UK, Coworking felt they had ‘started to get our head around some answers’ that they were more confident in sharing with politicians and policymakers. For instance, a leading Labour MP was involved in promoting Coworking.Union at their branch in her constituency.

However, the political connections that Union brought to the table did not enthuse everyone involved in Coworking, as one member commented:

They were members of a union. They were, as far as we could tell, on the right of the Labour Party. They were hostile to Jeremy Corbyn. And they had this agenda about self-employed people. I don't really know… how it all ties together, but it was just three observations that we made. And the other thing was… I think the impression was that it was a sort of old school unionist type of person, someone who was used to kind of fighting against other political ideas. […] I don't know how much you know about the UK Labour Party but, you know, it's a party which has got an awful lot of different schisms in it. So this was definitely part of a schism.

Conscious of some of these potential differences, Coworking ultimately aimed to use Coworking.Union to provide a platform to engage politically, while also providing a ‘good suite of services’ to members who did not want to participate in campaigning and advocacy. At times, however, participants from Coworking were clear that the collaboration with Union had emboldened Coworking to be more specific about what they offered next to other coworking space providers and organisations active in the self-employed workforce – in particular the ‘start-up culture’ that ‘tends to suck all the life out of spaces’ while they ‘become bigger and bigger’ – setting up a divide with independent workers who placed less emphasis on the more activist elements of their new organisational identity:

When we started working with the trade union, then it was clearer that we have political motivation and there were a few people, but not many, who would completely disengage from that… [But] we railed against that model that makes very wealthy people even wealthier… You know I think that if there are people who would disengage with that, who have no interest in what we're doing, but actually just want cheap workspace then they probably buy that coming here, they probably don't get the best out of us.

***Coworking.Union and Cooperative.*** The Coworking.Union partnership having been ‘already established’, Coworking continued their initial interactions with Cooperative by visiting the latter’s European headquarters in December 2017 along with representatives of Union. This was the first extensive contact between Coworking and Cooperative, and the first time Cooperative had met anyone from Union, after which the latter considered themselves as ‘working with’ Cooperative. As one Cooperative member described it,

We had a lot of exchange; they had a lot of questions on our model… And here we made them meet different people with different profiles. Because it's such a complex model, we thought it was important that they had different inputs. I can't remember exactly who they all saw, but they saw a bunch of people and it took a day and a half of encounters.

The meeting was generally deemed a success by the parties involved. Coworking came away persuaded of the potential impact it would have on self-employed workers in the UK, principally in its power to ensure that ‘people could get paid when they were putting their invoice across’. Moreover, Coworking saw Cooperative as representing the same ‘change the world’ mentality they had themselves.

Having met Coworking already, the European visit also enabled Cooperative to get to know Union. Having previously dealt with unions for whom it was ‘unbelievable that freelancers would have to pay for the employers’ contribution’, Cooperative were impressed by how ‘open minded’ the union seemed about their model, having ‘come from the [industrial] sector and transitioned to freelancing’ – a ‘huge leap’. With Coworking, Cooperative’s affinity was more natural, the two organisations sharing in common

a kind of a utopia of what work should be… because we're both working on the same type of population, let's say with freelancers, we envisage the same problems, but also kind of solutions ideally… So for us, people should all be able to develop their work from their skills and competencies, whatever, regardless of the legal status, they should have access to the same rights […] So there was this both the idea of what the problem is […] and how to bridge both economically and on the more social aspect.

The conversations over the course of the two days in the European capital city concerned the possibility and feasibility of exporting elements of the Cooperative model to the UK under the auspices of Coworking.Union. This began from a consideration of the potential difficulty of both Coworking and Union, as a coworking provider and trade union respectively, to ‘adapt to a model that is in between’ each, in a ‘grey zone’, and to make this work economically in the context of the underlying precariousness of coworking spaces and the contribution rates of union members. There was particular interest from Union in the technological element of Cooperative’s digital platform, the ‘optimisation’ of which for different kinds of contracts, benefits and statuses would be central to branching out into the UK context.

Another central topic was the practicality of internationalisation in the UK. Cooperative by this point was accustomed to the consideration of different opportunities to ‘adapt the model to different legal and cultural contexts’. The joint economic and social mission of the cooperative meant that many aspects of a nation’s political economy and industrial relations – the most important being the legal architecture governing a self-employed worker’s tax and employment status – were relevant variables in determining whether an internationalisation opportunity was worth pursuing in any given case. The outcome of this first proper meeting of the different parties was that Cooperative was ‘economically feasible’ in the UK, as well as legally. However, the specificity of ‘state organized social protection’ in the UK was seen as ‘not sufficient enough for the model to be interesting’. To create a business case, it would be necessary to do ‘more than mutualized aspects’, pushing the state to provide more rather than operating in the interstices of an existing system of ‘social contributions’ and protections.

Following the meeting at Cooperative’s European headquarters, Coworking.Union went away and continued developing their thinking about the possible form a business model would take in the UK and promoting the Cooperative model to policymakers and other stakeholders. While there was recognition that the ‘solution’ Cooperative posed to these issues ‘doesn’t necessarily work in the UK yet’, there was also the expectation that shifting political and economic conditions could enable the actors to start to ‘put some things in place’. This related in particular to one aspect of the Cooperative model. In the eyes of Coworking and Union, the main advantage of Cooperative’s ‘autonomous employer’ model was perceived to be the income-smoothing effect of being employed through an intermediary, whereby earnings could be pooled and salaries standardised on a monthly basis, overcoming income volatility and permitting access to benefits. The income-smoothing function of the Cooperative model was seen as a potentially attractive solution to this issue.

Further stimulus for exploration of the Cooperative model in the UK came from the enthusiasm of policymakers like Matthew Taylor, whom the government had tasked with a review of ‘modern working practices’ in 2017, and the Royal Society of Arts, which he led at the time, for a possible pilot scheme. Just as the legal grey area around self-employment had given Cooperative cover to expand in Northern European states, the same might be said for the weak regulatory environment in the UK. Something akin to the category of the ‘Cooperative worker’ might serve to bring much-needed clarity to the uncertain status of the self-employed for the purposes of equalising tax and National Insurance Contributions, for instance. Moreover, while the new Director of Labour Market Enforcement installed during the same period of the Taylor Review was tasked with their elimination, the often exploitative ‘umbrella companies’ used to manage payroll on behalf of temporary workers and agencies showed that there was precedent for intermediary bodies resembling Cooperative within the UK regulatory context without falling afoul of competition law on so-called ‘cartels’. In this apparently conducive policy context, Coworking engaged with politicians on both sides of the parliamentary divide, meeting government ministers and shadow cabinet members, finding little knowledge of the issues on the Conservative side and little interest on the part of the Labour Party in the battles of the self-employed.

After a period in which Coworking.Union explored and advocated for the Cooperative model domestically, there were renewed conversations with Cooperative, including a trip by a Cooperative delegation to Coworking’s headquarters in the UK (January 2018) and Coworking’s visit to Cooperative’s annual meeting in a European capital city (June 2018). For Cooperative, the former still felt ‘quite early’ in the relationship, it being ‘unclear’ what the options being explored would mean in practice, and the conversations were deemed more appropriate for a later stage ‘three years’ down the line rather than less than one year into the partnership.

### Dissolution and evolution

***Coworking.Union and Cooperative.*** The more Coworking.Union explored the possibility of rolling out the Cooperative model in the UK, the more difficult it seemed to be to envisage success. The main reason was that the model ‘doesn’t translate easily to the UK taxation system’, most notably the rates at which National Insurance Contributions (NICs) were paid. In the words of one participant,

the government in Westminster… doesn't know how to deal with self-employed people so it wants them to be paying the same taxes as the workers but to do that it needs to give them access to the same benefits…

On the basis that ‘just coming up with anything in a barren landscape is useful’, there was a period where some thought was given to how the Cooperative model could be part of the solution to this legislative impasse, and, as aforementioned, representations were made to the government and the Department of Work and Pensions. But the divergence between the tax and national insurance system in continental European countries (where Cooperative enjoyed a discounted rate of employer NICs because it employed so many people) and the UK ended up placing an unsurmountable barrier before attempts to think through the feasibility of a British version of Cooperative. A British Cooperative would have had to organise on the basis of an initially quite costly contribution by its members, but with no prospect of a discount once critical mass was reached. This is because, where NICs for the self-employed were around 9%, the combined contributions for employer and employee were 25.8%, a sum that Cooperative payroll would have to pay out of member subscription rates – the extra costs would be passed on to clients.

The legislative changes required to enable Cooperative to operate in the UK in a similar fashion as it did elsewhere were so great that little headway was made convincing policymakers, with the government proposing and later withdrawing legislation to redress the imbalance in contributions and protections. In this context, faith in the viability of the experiment dissipated. While perhaps not fully aware of the specificities of this policy stalemate, one interviewee sensed the cultural, political, and economic differences that placed the UK more in the ‘liberal’ orbit of the US than the more social or Christian democratic EU.

At the same time, whereas for Coworking political impossibilities prevented implementation of the Cooperative model, Cooperative went cold on the collaboration owing to more prosaic issues about how the relationship had developed. Cooperative was keen to stress that in order for a programme of internationalisation to work, they needed to be actively persuaded of the business case for doing so by the proposed partner. As one interviewee from Cooperative explained, this was a question of priorities:

We’re really not against it… But there's a difference between saying ‘why not’ and saying ‘let's do it’, and we're more at ‘why not’… If they're not going to propose something, we're not going to push it… I'm going to put into it and we can discuss financial aspects, but if we don't have that, we're not going to push it. We haven't had that really… except seeing each other and sharing a lot of values and enthusiasm and views and utopias even it was great but if they don't come to us with [something] we're not gonna push so well. So it's an open door but it's not a proactive when as I said internationalization is not necessarily a priority right now.

Coworking.Union failed to present the cutting-edge argument necessary to capture Cooperative’s sense of priority, expressing a reluctance to ‘replicate’ Cooperative and an acceptance instead that ‘they’re the experts and we just want to help them into the UK market’ – hardly the compelling business case the Cooperative team were hoping for.

To make matters worse, the relationship between Coworking and Union was breaking down. Union saw Cooperative as a ‘fantastic model’, despite the criticisms it had encountered from trade union counterparts in the very different European industrial relations context. Anticipating much less criticism from the labour movement in the UK, Union saw it as ‘a natural place for us to go’. However, despite Union’s ‘nimble and agile organisation’, there was felt to be insufficient institutional and governance support for the kinds of decisions necessary to develop the venture. The main driver of the project at Union departed the organisation. Had the driving force stayed, Cooperative would have been the ‘next step, the next logical thing for us to do’, according to a union official, instead the approach became ‘very transactional’ and support leaked from the endeavour. When Coworking and Cooperative met a final time at a Royal Society of the Arts event, one interviewee described the former as going through the process of a ‘very, very complicated’ breakdown of ‘trust’ with Union, that in turn brought an end to attempts to import Cooperative to the UK.

***Coworking and Union.*** The relationship between Coworking and Union disintegrated very quickly following the formation of Coworking.Union. As mentioned, the main point of contact and inspiration for the partnership at the union end left very soon after the deal was signed for Union to contribute a six-figure sum to Coworking. From Coworking’s perspective, the ensuing absence of other people with commitment to, understanding of, or care for the ‘output’ of the project put Coworking.Union ‘on a trajectory to fail’. The remaining union staff assigned to the partnership were perceived as being better suited to ‘someone who was unfairly dismissed’ or ‘had an industrial accident’, but less well-equipped to deal with ‘somebody who was earning next to nothing whose big guy wasn’t paying them their invoice in time’. According to one interviewee, this meant that they did not seem to ‘believe what we were saying about self-employed people being so precariously placed… I don’t think they were on the same page on this’. From Coworking’s perspective, this was bound up with politics:

So, the first seven years of our life were characterized with us never taking anybody else's money so it was all self-funded which meant that we could be completely honest with our worldview. As soon we have taken the union's money we were brought into a perspective of having to behave slightly differently and in reality I think that lost some of what Coworking was. Now in this world, sadly, you need money to survive so we've got to find ways… it's just picking the one that that actually works.

In this way, Union was seen as forcing upon Coworking a perspective that did not recognise workers or institutions that were not ‘affiliated or interested in party politics’, and were more concerned with ‘Labour leadership elections rather than worrying about whether [workers] get paid or not’. This imposition, in Coworking’s eyes, prevented them talking about some of the more radical ideas they were interested in politically, like the universal basic income or Breadfunds, an insurance scheme for the self-employed tested in the Netherlands. This was even seen as inhibiting Coworking’s ability to embrace the relationship with Cooperative. For Coworking, Union had become narrowly preoccupied with ‘selling a product’ – the range of services associated with Coworking.Union – of which Coworking’s leadership became a kind of ‘travelling salesman’ – ‘making a profit’ rather than ‘changing things’. Meanwhile, Union sometimes displayed an impression that Coworking’s ambitions were confined to coworking.

 These cultural differences and misunderstandings, while important, masked an important and intractable issue that undermined Coworking.Union: money. Once the main driver of the initiative had departed Union, the ‘vision’ was stripped back, and the underpinning material conditions of the collaboration began to play more of a structuring role. In particular, it was increasingly seen as ‘an exercise in growing membership’ alone, rather than the growth in membership supporting the construction of a broader and more independent alternative. Coworking had seen the experiment as ‘long term’, insofar as ‘you won’t see any benefits for those people for three, four, maybe even five years’. In line with their aforementioned ethos that ‘you can’t build a community, the community builds itself’, their intention was ‘connecting people’, without the expectation of an immediate benefit. But Union had invested a sizeable six-figure sum in the organisation and after two years ‘the membership figures didn’t match what they wanted’ in return for the amount they spent.

From Union’s perspective, the ‘membership density’ was not what they ‘would have liked’ and in that sense it was clear to the union that ‘the relationship didn’t work’ and the ‘agreement was finished’. The risks were clear to the union, insofar as the examples that had influenced the venture – such as the Freelancers Union in the US – while successful in some ways, had not engaged the self-employed workforce ‘at a significant level in terms of building membership density’. This being the key impetus for Union’s initial outreach into the grey zone of self-employment, ‘we could argue the same thing happened in the UK… the idea was good – it didn’t really get the attraction it maybe could have’. In Union’s view, they had helped Coworking ‘develop that model for two or three years’, but ‘Coworking had developed and moved on’ and the experiment had failed on their own terms.

Union was Coworking’s ‘biggest shareholder’, and when the decision was made to end the investment, a member of the union leadership called Coworking and, according to one participant, said ‘we’re gonna have to close you down then’. But, as a cooperative, Coworking was organised on a one-member, one-vote system, and Coworking’s response was ‘there’s another 1,000 people who’ve got votes as well, who may not want this to happen’. Later, after not turning up to the Coworking AGM, a representative of the union failed in their attempt to get voted onto the Coworking board, according to the same interviewee. The participant put these incidents down to a fundamental misunderstanding of ‘what it’s like to invest in a cooperative’, a ‘struggle to understand what a cooperative is’, and a difference in ‘values… even though cooperatives and unions were part of the same labour movement’.

That Union ‘decided to pull the plug without having invested all the money they expected to invest’ was described by one participant from Coworking as ‘hugely damaging’, leading to redundancies of key staff. Union ‘pulling out’ was thus experienced as a ‘dramatic event’ for Coworking, causing ‘huge difficulties’ that manifested in a long ‘struggle for Coworking to exist’ that has continued to the present day.

### Next steps

Coworking continued to offer the core ‘product’ associated with their collaboration with Union – insurance and legal support – but since the relationship ended with the union struggling to find investment sufficient to enable them to ‘market’ a ‘brand’ that was perceived as still having the potential to offer a lot of ‘value’ – language that showed shifting priorities from the values-driven project of previous years to one more concerned with the bottom line.

More broadly, in response to the financial situation that set in following the collapse of Coworking.Union, Coworking had begun to transform their business model to a more flexible, hands-off relationship with individual coworking spaces that attempted to ‘push down responsibility and try and encourage people to be independent’. In this spirit Coworking relied to a much greater extent on ‘anchor associates’ who ‘for a free desk manage the space so we don’t have to’. While in principle, however, Coworking was happy to hand as much money as required to someone who was willing to set up and operate a space, in practice they were finding that this was not easy ‘because people still think it’s cheaper to stay at home and be lonely and talk to the dog’. They were thus moving to a model where Coworking would rent desks at a space in which the organisation had no other ‘financial interest’, and then the website would simply point subscribing users towards those desks which they would be entitled to use out of their membership fees. This enabled both the ‘product’ and the space provision to coexist in a cross-subsidising fashion, without Coworking being exposed to the liabilities of taking on the running of spaces themselves.

But this low-resource model was not anticipated to be able to keep pace with the demands of resourcing existing coworking space operators and the ‘connectors’ that helped cohere those communities of self-employed workers. In this sense, it was widely accepted that if Coworking ‘is going to survive, it needs to survive in a different guise’ without the guarantee of any investment such as the large amount received from Union. Key personnel at the top of the organisation left and were replaced, with the view that new personalities would bring different and potentially more conducive approaches for new collaborations and partnerships in support of the organisation’s ambition to either ‘scale’ or ‘mature’. Meanwhile, the board of the cooperative had expanded in size from three or four people to 12, which was seen as a positive step forward.

However, while one interviewee had remained an ‘optimist’, they were ultimately ‘very pessimistic’. This was partly about the continued viability of operating coworking spaces, as the ‘audience’ for them had ‘changed quite a lot’. The participant was frustrated at how users ‘behave like children’ in expecting Coworking to ‘do everything for them’. But this pessimism extended more broadly to the political project the organisation pursued, and specifically with regards to the ‘chances of the self-employed in the UK’, whose status was ‘very weak’ and apparently unresolvable through the kind of collective action Coworking had attempted to pioneer. In practice,

Most of our members are running businesses, they're busy so they don't care sufficiently. They're pleased when we go and talk to politicians and persuade them of the argument that pleases them and some of them are pleased when they get the cheaper laptop… but does that mean that they get engaged in Coworking? … not really, so the reality is that very few people of the 500 engage in… the democracy of the organization which sadly means that actually most of the decision-making happens through [the leadership].

In line with this downbeat appraisal, Coworking expressed no desire to ‘resolve the relationship with Union’ on the basis that they did not ‘think it matters at all to the people we’re trying to help’. Union, for their part, had taken the experience of working with Coworking as a ‘learning curve’ that paved the way for them to eventually explore or realise partnerships with other organisations active in the representation of non-standard workers operating in the grey zone of employment – unemployed and underemployed as well as self-employed. On hearing that Union had since gone on to work with a much longer-standing association of independent professionals and self-employed workers, Coworking were keen to highlight that the latter ‘work with people who are earning sixty, seventy, eighty thousand pounds a year, they’re not impoverished’ – unlike the perceived target audience of the cooperative.

As regards Cooperative, on the other hand, Coworking expressed an ‘openness’ to work with them again in the future. Coworking continued to believe that the implementation of the model in the UK would be ‘a huge opportunity if someone got it right’ because of the persistent difficulties faced by self-employed people in accessing mortgages, for instance, owing to volatile pay. While the question of the incompatibility of the Cooperative model with the UK taxation system remained to be resolved, there was a belief that something like Cooperative could still be a potential solution to the impasse in Westminster. The feeling was mutual, insofar as Cooperative said they ‘really want[ed] to continue to work with Coworking’, even though they were reluctant to ‘go chasing it right now’ and would rely on the UK partners coming up with a ‘reliable model’ for doing so.

While sustaining a slightly less direct relationship with Cooperative, participants from the Union side also remained positive about the possibility of bringing Cooperative to the UK. One participant saw the changing economic climate in the UK post-Brexit meaning an increase in independent or self-employed work that would leave ‘a vacuum… that can be filled with a thoughtful organisation that’s prepared to invest some time and resources and take a long-term view on this’. Indeed, it was participants connected with Union who seemed most optimistic about the potential to organise the self-employed in the UK, and specifically in those contexts where ‘freelancers work collectively’ such as broadcast and construction and ‘traditional organising models’ familiar to the repertoire of trade unions would still be applicable: recruitment, health and safety reps, individual and collective representation. However, it would still be necessary to make these workers an ‘offer’ of value and relevance to them:

whether it's factoring arrangements to make sure they get paid on time, whether it's providing some form of access to portable benefits, whether it's providing access to a coworking space when they need to, whether… it's bringing these people together so they can collaborate and go together and get work together…

The interviewee saw trade unions and cooperatives still playing an important role in such an offer, possibly in collaboration with coworking spaces. However, it would require two key factors: ‘finding credible partners to work with to help you deliver for people who need professional services’, possibly including private sector providers of benefits and support; and ‘finding campaign issues that are important and being their voice’. It was thus implied that one of the reasons Coworking.Union did not succeed was for a lack of these two factors.

## Discussion and conclusions

In this paper, through long-term ethnographic work conducted in parallel by the authors and through semi-structured interviews carried out in a coordinated manner, we analysed a collaboration involving three key actors in the context of self-employment: Union, Coworking, and Cooperative. The study of each of the three organisations mobilises specific literatures that only occasionally intersect. The contribution of our case study is the dialogue it constructs between the three reference literatures, highlighting their combined relevance for our understanding of the opportunities and limitations of organising the self-employed.

In the preceding section, we presented a detailed reconstruction of the interactions of the three actors over time, highlighting the reciprocal positioning of the three players. This covered the context, expectations, and visions of the three actors and their evolution over time, starting from the motivations that generated the first contacts through to the development of operational agreements and up to the failure of these agreements as relations between them cooled.

The case study, and the failed experiment it captures, constitutes an important opportunity to understand the dynamism, complexity, and contradiction manifest in organising the self-employed, of which the case is emblematic. While the strategic ingredients of significant organisational innovation were in evidence between the three actors, it generated instead a failure from which useful lessons may be nonetheless drawn. From this point of view, therefore, the case study demonstrates the importance of an in-depth articulation and analysis of failed attempts at organising the self-employed and their meaning for broader struggles by old and new actors to alter the terrain of the ‘grey areas’ of employment more generally. In particular, the case study contributes to continuing attempts to conceptualise alternative modes of organising by representing a unique combination of three distinct kinds of experimentation in a common endeavour, in turn exposing the paradoxes and contradictions that characterise them individually.

The case took place in the context of a national institutional structure that provided a weak response to the emerging need for new forms of regulation and protection fit to address the changes, conditions and challenges faced by a growing self-employed workforce. Into this partial institutional vacuum stepped the bottom-up initiative promoted by Coworking and Union, including the attempted adaptation of the Cooperative model in the UK. The idea of combining the competences of the three actors seemed promising from the outset: Coworking was developing a coworking network in brownfields and rural areas to offer a sustainable alternative solution to the concentration of the self-employed in large urban areas. The trade union Union, for its part, offered considerable experience in collective representation and, by virtue of its interest in exploring the context of self-employment, a significant budget to invest in collaboration with strategic partners. Cooperative, meanwhile, was the authoritative guarantor able to offer long-standing organisational experience in the cooperative and mutualist frame for self-employed workers from different sectors, and the provision of a model that could inspire Coworking.Union.

This combination of actors possessed significant political and organisational potential to provide a voice for self-employed workers while also materially advancing the capacity to represent and bargain for self-employed workers in the labour market and beyond. The findings demonstrate that the different actors brought with them different expectations of what the experiment would achieve. For Coworking, the desire to create a political voice for the self-employed motivated their involvement. While certain elements within Union had the same desire, such a role would have principally served the end of expanding membership and bargaining capacity in order to grow the union, rather than achieving an end in itself. The expectations different actors had constructed around these two possibilities increasingly came into conflict with one another. Union was committed to the political objectives only insofar as they corresponded with the union’s understandable priority to increase its power and presence in exchange for the investment of members’ subscription funds. Coworking, meanwhile, perceived the constraints on the union’s engagement in the experiment as expressing an instrumental approach that limited what the collaboration could achieve. This mismatch of expectations constrained the capacity of Coworking.Union to provide a persuasive and feasible model for the implementation of the Cooperative model in the specific institutional context of the UK, leading to the eventual end of the collaboration.

In these respects, the two key actors in the attempted experiment remained confined, often understandably, to their existing set of practices. Beholden to their specific organisational logics, they failed to create an adequate or durable institutional carrier for a new approach to representation and decision-making. The case study thus complicates claims that there is anything inevitable about the emergence of new actors and new alliances to represent the unrepresented in the ‘grey areas’ of employment. Such new forms of representation require concerted organisation to be achieved, and concerted institutionalisation to be sustained – especially, as in this case, where the existing institutional environment of the specific national political economy makes experimentation difficult due to a weak regulatory context.

At the same time, the specificities of the different actors involved in the case study – unions, coworking spaces, cooperatives – demand that we recognise the different opportunities for bargaining, representation, and organisation that they offer different groups of workers, particularly the self-employed. One response to the emergence of the grey area of new working practices is to identify the workers that inhabit it with a pervasive condition of precariousness within which specificity is ironed out and all struggles are articulated together. Nevertheless, this case concerns a workforce and a set of organisational actors that already carry their own specific identities and goals. Rather than a foundational commonality, the experiment was characterised by an original plurality that reflects the complexity of contemporary labour and contemporary society. Any unity of purpose would be established through organisational experimentation rather than automatically, through the setting of common goals and creation of common identities. The construction of ‘solidarity in difference’ ( Fleischmann *et al.*, 2022) cannot but imply that such a collaboration is by necessity experimental precisely because of the possibility of failure that haunts its attempts to effect change. In this sense, the value of an attempt such as the one studied lies in having explored an articulated and complex pathway that seeks to bring together the cornerstones of labour and workers’ organisation with, for instance: democracy, through cooperativism and mutualism; collective representation, through trade unions; and collective organisation of labour, through the infrastructures that a politicised model of coworking can offer.

The question remains, of course, whether new and old actors need to unite under a single banner in order to articulate specific struggles, or whether it is better that different groups of workers and the particular positions they occupy within labour markets are addressed and satisfied through different organisational and institutional means. In other words, whether trade unions with traditional structures or cooperatives with more radical forms of representation and decision-making can intertwine their action and structures. What the case study actually suggests is the specificity of different economic and political positions and the specificity of the organisational and institutional structures that have, and must, spring up around them in order to address the particular circumstances of different groups. There will inevitably continue to be contradictions between the need for a trade union like Union to ensure value for money in prioritising the representation of its members and the need for Coworking to grow its membership through more flexible means, for instance. These contradictions could prove to be productive, harnessed in a spirit of open-minded organisational experimentation. But ultimately, these different characteristics provide tailor-made organisational responses to the specific needs of the workers they address. Were everything to be brought under one institutional roof, the fragility of such an experiment may risk the capacity of the organisations involved to service the basic needs their members or audiences demand to be satisfied. In this sense, it is enough for traditional unions to do their thing, and for new organisations for the self-employed to do theirs, without the pursuit of ‘one big union’. As our case study shows, this may actually serve to weaken rather than strengthen nascent experiments, subordinating them to logics that constrain their capacity to innovate.

## Ethics and consent

Written informed consent for publication of the participants' details in pseudonymised or aggregated form was obtained from the participants.

The research project SHARE has been reviewed and given a favourable opinion from:

- The ERC ethical review board on 22/02/2017-Ref. Ares(2017)960489;
- The Research Ethics Committee of the University of Leeds on 13/09/2017, ethics reference LTLUBS-175;
- The Research Ethics Committee of the University of Milan on 08/11/2018, ethics reference No. 50/18;
- The Data Protection Officer of the University of Milan on 10/11/2018;
- The ERC ethics officer approved all the new elements submitted for ethics on 28/05/2019.

## Data Availability

The ERC project SHARE was granted in 2016 and the PI did not opt into Open Research Data, as at that time it was a pilot and data collection in comparative qualitative is extremely complex. The underlying data for this project therefore cannot be shared.

## References

[ref-1] Almond P Connolly H : A manifesto for ‘slow’ comparative research on work and employment. *European Journal of Industrial Relations.* 2020;26(1):59–74. 10.1177/0959680119834164

[ref-2] Bajard F Leclercq M : Devenir entrepreneur(e) en coopérative d’activité et d’emploi. *J Soc Am.* 2019;158-159:151–174. 10.4000/jda.8834

[ref-3] Borghi P Murgia A Mondon-Navazo M : Mind the gap between discourses and practices: Platform workers’ representation in France and Italy. *European Journal of Industrial Relations.* 2021;27(4):425–443. 10.1177/09596801211004268

[ref-40] Bozzon R Murgia A : Independent or dependent? European labour statistics and their (In) ability to identify forms of dependency in self-employment. *Soc Indic Res.* 2022;160(1):199–226. 10.1007/s11205-021-02798-1

[ref-4] Burchell B Deakin S Honey S : The Employment Status of Individuals in Non-Standard Employment.Department of Trade and Industry, Employment Relations Research Series No. 6, DTI London,1999. Reference Source

[ref-5] Bureau MC Corsani A : The French Business an Employment Cooperative. An autonomy factory?In: Emi Armano, Arianna Bove, Annalisa Murgia (Eds.) *Mapping Precariousness: Labour Insecurity and Uncertain Livelihoods.* London: Routledge,2017;60–69. 10.4324/9781315593838-6

[ref-6] Buschoff KS Schmidt C : Adapting labour law and social security to the needs of the'new self-employed'—comparing the UK, Germany and the Netherlands. *J Eur Soc Policy.* 2009;19(2):147–159. 10.1177/0958928708101867

[ref-7] Butcher T : Learning everyday entrepreneurial practices through coworking. *Management Learning.* 2018;49(3):327–345. 10.1177/1350507618757088

[ref-8] Conaty P Bird A Ross C : Working Together. Trade union and co-operative innovations for precarious workers. 2018. Reference Source

[ref-9] Conen W Schippers J, (eds.) : Self-Employment as Precarious Work: a European perspective.Cheltenham: Edward Elgar,2019;296. Reference Source

[ref-10] de Peuter G Cohen NS Saraco F : The ambivalence of coworking: On the politics of an emerging work practice. *Eur J Cult Stud.* 2017;20(6):687–706. 10.1177/1367549417732997

[ref-11] Fleischmann A Holck L Liu H : Organizing solidarity in difference: Challenges, achievements, and emerging imaginaries. *Organization.* 2022;29(2):233–246. 10.1177/13505084221083907

[ref-12] Gandini A Cossu A : The third wave of coworking: ‘Neo-corporate’ model versus ‘resilient’ practice. *Eur J Cult Stud.* 2021;24(2):430–447. 10.1177/1367549419886060

[ref-13] Grazian D : Thank God it’s Monday: Manhattan coworking spaces in the new economy. *Theor Soc.* 2020;49(5):991–1019. 10.1007/s11186-019-09360-6

[ref-14] Heery E : Partnership versus organising: alternative futures for British trade unionism. *Industrial Relations Journal.* 2002;33(1):20–35. 10.1111/1468-2338.00217

[ref-15] Houtbeckers E : Framing social enterprise as post-growth organising in the diverse economy. *mrev management revue.* 2018;29(3):257–280. 10.5771/0935-9915-2018-3-257

[ref-16] Hyman R Gumbrell-McCormick R : Resisting labour market insecurity: Old and new actors, rivals or allies? *J Ind Relat.* 2017;59(4):538–561. 10.1177/0022185617714423

[ref-17] Ibsen CL Tapia M : Trade union revitalisation: Where are we now? Where to next? *J Ind Relat.* 2017;59(2):170–191. 10.1177/0022185616677558

[ref-18] Joyce S Stuart M Forde C : Theorising labour unrest and trade unionism in the platform economy. *New Technology, Work and Employment.* 2022;38(1):21–40. 10.1111/ntwe.12252

[ref-19] Lockey A : Free Radicals.London: Royal Society of the Arts,2018.

[ref-21] Martinelli F Bozzoni S Caroli S : Platform cooperativism in Italy and in Europe.(No. 27). CIRIEC International. 2019. Reference Source

[ref-22] Meardi G Simms M Adam D : Trade unions and precariat in Europe: Representative claims. *European Journal of Industrial Relations.* 2021;27(1):41–58. 10.1177/0959680119863585

[ref-23] Merkel J : ‘Freelance isn’t free.’ Co-working as a critical urban practice to cope with informality in creative labour markets. *Urban Studies.* 2019;56(3):526–547. 10.1177/0042098018782374

[ref-24] Mollona M : Community unionism versus business unionism: The return of the moral economy in trade union studies. *American Ethnologist.* 2009;36(4):651–666. 10.1111/j.1548-1425.2009.01201.x

[ref-25] Mondon-Navazo M Murgia A Borghi P : In search of alternatives for individualised workers: A comparative study of freelance organisations. *Organization.* 2022;29(4):736–756.

[ref-27] Murgia A Bozzon R Digennaro P : Hybrid Areas of Work Between Employment and Self-Employment: Emerging Challenges and Future Research Directions. *Front Sociol.* 2020;4:86. 10.3389/fsoc.2019.00086 33869406 PMC8022784

[ref-28] O’Grady F Nowak P : Beyond New Unionism.In: P Willman and J Kelly (eds.) *Union Organization and Activity.* London: Routledge,2004;150–163. Reference Source

[ref-29] Olsen KM Kalleberg AL : Non-standard work in two different employment regimes: Norway and the United States. *Work Employ Soc.* 2004;18(2):321–348. 10.1177/09500172004042772

[ref-30] Pitts FH : ‘A science to it’: flexible time and flexible subjectivity in the digital workplace. *Work Organ Labour Glob.* 2013;7(1):95–105. 10.13169/workorgalaboglob.7.1.0095

[ref-31] Pitts FH : Rhythms of Creativity and Power in Freelance Creative Work.In: *Virtual workers and the global labour market.*eds. K. Randle and J. Webster. Basingstoke: Palgrave,2016;139–159. 10.1057/978-1-137-47919-8_7

[ref-32] Pitts FH : Creative Labour, Before and After ‘Going Freelance’: Contextual Factors and Coalition-Building Practices.In: *The new normal of working lives: Critical studies in contemporary work and employment.*eds. Stephanie Taylor and Susan Luckman. London: Palgrave Macmillan,2017;87–107. 10.1007/978-3-319-66038-7_5

[ref-33] Pitts FH : Creative Labour, Metabolic Rift and the Crisis of Social Reproduction.In: *Cultural Industries and the Environmental Crisis.*eds. Kate Oakley, Mark Banks. Cham: Springer,2020;111–124. 10.1007/978-3-030-49384-4_9

[ref-34] Spreitzer GM Cameron L Garrett L : Alternative Work Arrangements: Two Images of the New World of Work. *Annu Rev Organ Psychol Organ Behav.* 2017;4(1):473–499. 10.1146/annurev-orgpsych-032516-113332

[ref-35] Supiot A : Beyond employment. Changes in work and the future of labour law in Europe.Oxford: Oxford University Press,2001. Reference Source

[ref-36] Symon G Crawshaw J : Urban labour, voice and legitimacy: economic development and the emergence of community unionism. *Ind Relat J.* 2009;40(2):140–155. 10.1111/j.1468-2338.2008.00517.x

[ref-37] Taylor M : Good work: the Taylor review of modern working practices.London: Department for Business, Energy & Industrial Strategy,2017. Reference Source

[ref-38] Waterman P : ‘Social Movement Unionism’ in Question: Contribution to a Symposium. *Empl Responsib Rights J.* 2008;20(4):303–308. 10.1007/s10672-008-9093-z

[ref-39] Wills J : Community unionism and trade union renewal in the UK: moving beyond the fragments at last? *Trans Inst Br Geogr.* 2001;26(4):465–483. Reference Source

