# Supplementary material for: Organising the self-employed: combining community unionism, coworking and cooperativism across contexts
Source: Open Res Eur. 2023 May 16;3:80. [Version 1] doi: 10.12688/openreseurope.15798.1 (PMC10483176; doi:10.12688/openreseurope.15798.1)
Supplement: Peer review history

# Peer review: Reviewer response for version 1

**DOI:** 10.21956/openreseurope.17065.r34421

**Published:** 2023-09-06

**Authors:** Aurora Trif

**Affiliations:** 1 DCU Business School, Dublin City University, Dublin, Leinster, Ireland

This case study contributes to a better understanding of the complexities and struggles to develop collective representation for workers on non-standard contracts. It provides a comprehensive description of a failed collaboration between a trade union and two new collective actors in representing and organising self-employed creative workers. This case covers key aspects concerning the three actors involved, the development of the relations amongst them within the given context and, outcomes of their main actions. Although it is very informative and well written, at times, it is difficult to follow the overall argument. For instance, could it be argued that is impossible for trade unions to collaborate with other organisations to represent the collective interests of the growing number of self-employed professionals? If not, under which conditions such collaboration is more likely to succeed in improving working conditions for self-employed based on your findings?

 First, it would benefit from a stronger conceptual and analytical foundation. As regards theoretical underpinning, it could draw on union revitalisation literature. If so, it could contribute to answering whether is possible for trade unions to collaborate with other organisations to represent the collective interests of the growing number of workers on non-standard contacts. Nevertheless, the authors could to draw on other literature and develop a different overarching question that would make it easier for the readers to follow their argument. As regards the analytical aspects, the paper could be more explicit about what is meant by success or failure in this case study. It is specified that there was an “attempt to create a new institution”. Still, success could also mean any joint action undertaken by two or three actors that improved any working conditions for self-employed.

 Second, the key aspects examined in this case study could be presented in a simpler format by using tables (or figures), while providing less details in the text. Please see below some suggestions:

 e.g. Table 1 – Key characteristics of each actor*

 The following aspects might be covered for each actor: *Set up *(year/decade), *Purpose,* *Key constituents /target group, Size (*number of members) *& Respondents (*number).

 *Minor point: I would suggest to amend the pseudonyms of actors (e.g. maybe add 1-2 capital letters at the end, such as SE), to make it easier for the reader to differentiate whether you refer to the ‘union’ or ‘cooperative’ as a generic term or case study actors).

 Table 2: Key characteristics of the development of relations among the three actors over time. It might cover key joint actions (bipartite and/or tripartite) in Stage 1, Stage 2 & Stage 3, for instance.

 Table 3: Antecedents of successful and/or unsuccessful outcomes of joint (bipartite & tripartite) actions actors over time.

 Table 3 could be useful to identify and discuss under which conditions such collaboration is more likely to succeed in improving working conditions for self-employed. Also, it could help with identifying practical implications. Specifically, what are the lessons learnt from this failed experiment for trade unions, cooperatives and coworking organisations seeking to further the interests of self-employed as well as for policy makers?

 Finally, it might be useful to provide more details concerning the research methodology, if possible without compromising the anonymity. While the paper presents relevant findings and very interesting quotes, it could provide further details about interviews (e.g. how many representatives of each actor were interviewed? Were these respondents interviewed multiple times? If so, when – e.g. stage 1, 2, etc?) and maybe the number of meetings attended at each stage.

 Overall, this is a very interesting case of a failed collaboration between trade unions and other new collective actors that would be of particular interest to policy makers and collective actors representing the interests of workers on non-standard contracts.

Is the case presented with sufficient detail to be useful for teaching or other practitioners?

Yes

Is the work clearly and accurately presented and does it cite the current literature?

Partly

If applicable, is the statistical analysis and its interpretation appropriate?

Not applicable

Are all the source data underlying the results available to ensure full reproducibility?

No

Are the conclusions drawn adequately supported by the results?

Yes

Is the background of the case’s history and progression described in sufficient detail?

Yes

Reviewer Expertise:

Industrial relations, precarious work, trade union revitalisation

I confirm that I have read this submission and believe that I have an appropriate level of expertise to confirm that it is of an acceptable scientific standard, however I have significant reservations, as outlined above.

# Peer review: Reviewer response for version 1

**DOI:** 10.21956/openreseurope.17065.r31690

**Published:** 2023-09-06

**Authors:** Flora Bajard

**Affiliations:** 1 LEST - Laboratoire d'Economie et de Sociologie du Travail (UMR 7317), CNRS (Centre National de la Recherche Scientifique), Marseille, Provence-Alpes-Côte d'Azur, France

Taking as a case study an original experiment in organizing the self-employed, this article outlines the main drivers and limits of new modalities of mobilization, in a context of gray-zones of employment and increasingly complex work situations. This attempt takes place at the intersection of three organizations and two social-economic and legislative spaces: 'Union', a historic trade union which, in the early 2000s, began to turn its attention - through community unionism - to new groups initially excluded from union representation, such as the self-employed; 'Coworking', a co-working and interest representation organization for the self-employed founded in 2010 (both are located in the UK); 'Cooperative', a freelancers' company founded in the late 1990s which expanded in Western Europe during the 2010s and is based in a European capital city. Drawing on the strengths offered by these three organizations, a new one was created: 'Coworking. Union', supposedly integrating the experience in collective and budget representation on the one hand, and the experience in the mutual and cooperative organization of freelancers and knowledge of their issues on the other hand.

 Through a **longitudinal study of these three organizations **- between 2017 and 2021 - **this article exposes the ins and outs of what turned out to be a failure**. In short, **it successfully inverts the now classic question of "unlikely mobilizations"**, which proposed to understand how causes had emerged in counter-intuitive ways. Instead, the article takes as its central axis the following paradox: how can we explain the failure of this initiative when it was deployed in a context of significant and urgent need for new forms of representation and protection for this emerging workforce, i.e. the self-employed?

 By bringing to light the contradictions and divergences of visions and interests (with, for example, some highly interesting developments on both Union and Coworking's relationship to politics, p. 10) as well as other difficulties (e.g. the tax and accounting issues, p. 10), the article develops a major posture that the sociology of collective action had initiated through the theory of resource mobilization: **setting collective actors in motion is never self-evident. This common approach now in political sociology, appears salutary when applied to the field of study of employment and work**: indeed, its growing complexity is leading some researchers to consider the inescapable character of the "representation of unrepresented" (p. 13). However, it is essential to remind, as this article does, that transformations in labour relations and markets does not automatically generate effective transformations, far beyond the media discourses about inexorable social innovations.

 This is **another strong point of this article, which also takes up certain proposals aimed at positioning interest groups and social movement actors on a continuum**. Even if such an interweaving is often invoked in literature, it however remains little practiced in the social sciences, still too compartmentalized between the sociology of industrial relations and trade unionism, the sociology of interest groups and, finally, the sociology of work centered on cooperativist movements and workers' self-organization. As pointed out on p. 12, by positioning itself at the heart of these different subfields, **this research raises important issues, both for the scientific literature and for the actors** of social change:

- What common issues and strategies could enable these different organizations to be thought of in a common way, beyond their apparent separation (e.g. search for representativeness and extension of their base, financial resources, effectiveness of lobbying and advocacy, mediatization of certain issues...)? This is by the way one of the intellectual 'tricks' given by H.S. Becker, ***to link together different social phenomena that apparently belong to different spheres***, to make some general and shared dynamics appear (Becker 2016). ^1^ 
- How are ***the specific interests of certain social groups -*** or segments of groups - ***combined with broader and cross-cutting interests***? This tension, mentioned in the conclusion (p. 15), raises the strategic challenge of reconciling the differences and singularities emerging in today's labor markets with their possible rallying around generic watchwords, such as "democracy", "collective representation or organization" or "precariousness" (p. 14). Overall, this appears to be a useful lesson for the sociology of social movements, far beyond work and employment issues.

Ultimately, this article reveals a dual movement: on the one hand, a diversification of interests due to the increasing complexity of the world of work; on the other, the possible construction of lines of analysis and dynamics that cut across different social spaces. Thus, beyond the self-employed, articles like this one **invite us to draw lines of transversality to understand certain social realities**, even when the objects have been assigned to different types of literature due to the history of the constitution of our disciplines, as well as the social frontiers in our contemporary world.

 In line with this idea of the complexification of strategies and interests, I would now like **to propose a few suggestions** to the authors:

 a) It seems to me that the article might benefit from **making these strategic stakes more visible in the expression of its core issue**; in particular, in terms of the recomposition of social classes, the representation of the different groups that make up the worlds of work, and economic or relational strategies with certain segments of the public policies. As it stands, the text mainly pretends to bring together different streams in the literature and only mentions the interest of studying " *an attempt to create a new institution from elements of each* [organization]" (p. 5) without specifying further with what stakes; in other words, the text seems to set out its contributions too modestly.

 b) Generally speaking, the article seems to focus on the **worldviews and practices of the leading segment of the three organizations** (representatives, spokespersons and those responsible for missions within them), even though it is specified that ethnographic observations were carried out in collective moments including a large proportion of the members (p. 5).

- This point would benefit from methodological clarification, for example by ***specifying the social scenes observed*** (we only know that these were " *meetings and other events involving representatives*", p. 5) ***and the roles or functions assumed by the interviewees*** with whom the six semi-structured interviews were conducted.
- With this in mind, I also suggest - for instance, by providing some details about the type of profession, role or function assumed, length of time in position - ***limiting as far as possible formulations that leave the place of the speakers undecided***, such as: " *one cooperative member*" (p. 8), " *one participant*" (p. 11), " *participants*" (p. 12), " *certain elements within Union*" (p. 13), etc. Similarly, we encourage authors to systematically specify the origin of quoted statements: e.g. p. 6 (" *there were people in Union*..." and following sentences), p. 7 (" *showing return on [their] investment*..."), p. 8 (" *a kind of a utopia*..."). [« *a kind of a utopia of what work should be… *» p. 8 (at the bottom, last paragraph of the page)]
- Finally, this approach can also appear in the clarification of certain ***details concerning the positions quoted, or observations made within the organizations***: are certain speeches the reflection of collective positions established after a deliberation process, or rather a dissenting confidence of certain members to the researcher(s)?

In short, while guaranteeing the anonymity of the respondents, such details would make it possible to relate the positions taken to social positions, and thus **to further sociologize the analysis of this organizational complexity. In so doing, it would also dispel any grassroots myths or illusions of internal homogeneity** within these organizations (whereas, as A. Strauss pointed out (Strauss 1992 ^2^), they are no more than merely aggregates of segments, sometimes agonistic). Thus, the article is not necessarily intended to deal with the complexity of strategic arrangements applied within organizations, precisely because of the complexity of this dimension and the need to limit the article to a single point. However, it would make the existence of this additional challenge explicit.

 c) Lastly, two essential conditions for the realization of such mobilization projects are alluded to in an all-encompassing way, with the ideas of " *concerted organisation to be achieved*" and " *concerted institutionalisation to be sustained*". The various organizational and strategic interests exposed in the article are actually distributed between these two broad categories, and are very well detailed in the evolutionary restitution of the ideals and developments of the project, right up to its failure. However, **the economic and materialistic dimension, which is a central issue (p. 11), might deserve more detail** in p. 3-4-5, for example by providing economic data on each of the organizations: budget, number of salaried employees, etc.

 d) Finally, and most prospectively, I encourage the authors **to develop further in the future one of this work's strengths**, mentioned briefly in the conclusion: the **benefits of integrating the analysis of social experimentation into long-term processes. **The article thus evokes the factors that " *weaken rather than strengthen nascent experiments*" (p. 14), and it is likely to be an interesting path to study the future impacts of such a failure within the development of each of the organizations; more generally for social science, it is a point of interest to draw the attention to the longitudinal life of social entities.

**References**

1. Becker, H. (2016). I / Aller voir ailleurs : raisonner sur le monde à partir d’un cas. Dans : , H. Becker, *La bonne focale: De l'utilité des cas particuliers en sciences sociales* (pp. 15-55). Paris: La Découverte.
2. Strauss A.L., 1992. – *La trame de la négociation. Sociologie qualitative et interactionnisme*, Paris, L’Harmattan.

Is the case presented with sufficient detail to be useful for teaching or other practitioners?

Partly

Is the work clearly and accurately presented and does it cite the current literature?

Yes

If applicable, is the statistical analysis and its interpretation appropriate?

Not applicable

Are all the source data underlying the results available to ensure full reproducibility?

Partly

Are the conclusions drawn adequately supported by the results?

Yes

Is the background of the case’s history and progression described in sufficient detail?

Yes

Reviewer Expertise:

Sociology of work and employment ; political sociology ; sociology of professions ; sociology of arts and culture ; qualitative methods

I confirm that I have read this submission and believe that I have an appropriate level of expertise to confirm that it is of an acceptable scientific standard.

## References

[1] : La bonne focale.2016; 10.3917/dec.becke.2016.01 10.3917/dec.becke.2016.01

[2] : La trame de la négociation: sociologie qualitative et interactionnisme. *L'Harmattan Paris* .1992;

# Peer review: Reviewer response for version 1

**DOI:** 10.21956/openreseurope.17065.r33073

**Published:** 2023-08-02

**Authors:** Nathan Lillie

**Affiliations:** 1 Department of Social Sciences and Philosophy, Faculty Member, University of Jyväskylä, Jyväskylän yliopisto, Finland

The growth of self-employed creative and knowledge work presents a fundamental challenge to existing systems of worker representation, and this case study illustrates quite clearly why this is the case. It is also an interesting case study illustrating barriers to interorganizational cooperation. Different kinds of organizations have different strengths and weaknesses, which motivates cooperation, but this fact also can scupper cooperation, as the case study shows. Union and Cooperative were well paired because of their differences, but those differences also undermined the cooperation It appears from the narrative that they required someone able to work in both worlds in order to make the cooperation work.

 The case narrative is well researched and empirically rich. It is well written and interesting, and raise various important issues. However, the manuscript is theoretically weak, and therefore does not reach its potential. The literatures it engages with on community unionism, coworking spaces and cooperatives, are appropriate to engage with but more or less simply set out the problems these organizations attempt to address and the solutions they present. Obviously, these things should be discussed and form part of the analysis, but don't provide an analytical framework to generalize the analysis. Since this case is being presented as a case of organizational cooperation (there are other ways the authors could go with this, but interorganizational cooperation is a good option), I suggest using a theory that deals with such matters to frame the analysis: institutional entrepreneurs, bridge builders, identity work, co-production (there are probably other options as well). This would mean cutting back a little on the discussion of the other literatures, but it seems to me like some of that could go without any loss to the analysis. 

 Another issue is, how is this ethnography? It seems like a pretty conventional case narrative; the data collected is good and detailed, which implies a decent level of access which was no doubt facilitated by ethnographic techniques to gain access. That's fine, as far as it goes, but the authors seem to have written themselves out of the story. This is not a serious flaw, as a good case study is still a good case study, but more of a missed opportunity to add texture and depth. If there is the data, some descriptions of direct observations involving work processes, disputes and issues of individual workers, and daily interactions to show community building (or not) would be interesting, although I recognize words used here would have to be balanced against description of the organizational governance and cooperation processes in terms of analytical value. I would say the manuscript could be published as is, but would benefit greatly from a stronger theorization, drawing on concepts of organizational identity, and synergies. The authors might also consider what they can do to give it a stronger ethnographic texture and feel.

Is the case presented with sufficient detail to be useful for teaching or other practitioners?

Yes

Is the work clearly and accurately presented and does it cite the current literature?

Partly

If applicable, is the statistical analysis and its interpretation appropriate?

Not applicable

Are all the source data underlying the results available to ensure full reproducibility?

No

Are the conclusions drawn adequately supported by the results?

Yes

Is the background of the case’s history and progression described in sufficient detail?

Yes

Reviewer Expertise:

Social and public policy, industrial relations

I confirm that I have read this submission and believe that I have an appropriate level of expertise to confirm that it is of an acceptable scientific standard, however I have significant reservations, as outlined above.
